# Use of continuous infusion of nicardipine to control persistent postpartum hypertension: A retrospective study

**DOI:** 10.1097/MD.0000000000032381

**Published:** 2022-12-23

**Authors:** Min Kyung Kim, Ki Roong Choe, Da Eun Jeong, Kyong-No Lee, Iseop Cho, Hyeon Ji Kim, Jee Yoon Park

**Affiliations:** a Department of Obstetrics and Gynecology, Seoul National University College of Medicine, Seoul National University Bundang Hospital, Gyeonggi-do, Republic of Korea; b Department of Computer Science and Engineering, Seoul National University, Gwanak-gu, Seoul, Republic of Korea.

**Keywords:** nicardipine, continuous infusion, persistent hypertension, postpartum

## Abstract

To evaluate the effect of continuous infusion of nicardipine on the management of uncontrolled blood pressure (BP) during postpartum period. This retrospective study included 209 women diagnosed in hospital with hypertensive disorders during pregnancy and had uncontrolled BP after delivery between January 2018 to December 2020 Uncontrolled BP was defined as persistent elevation of systolic BP ≥ 160 mm Hg or diastolic BP ≥ 110 mm Hg. Patients were divided into 2 groups: nicardipine (N = 53; continuous nicardipine infusion and additional bolus of labetalol or hydralazine) and control (N = 156; consecutive bolus of labetalol or hydralazine). BP data were analyzed using the Mann–Whitney *U* and *χ*^2^ tests by dividing the time interval of 4 hours by the delivery time. The highest BP trends showed that the mean values of both systolic and diastolic BP immediately before delivery were higher in the nicardipine group than in the control. After 8 to 12 hours following delivery, both systolic and diastolic BP were lower in the nicardipine group than in the control. Subsequently, 16 to 20 hours after delivery, both systolic and diastolic BP were significantly lower in the nicardipine group than in the control (137/80 vs 141/84 mm Hg). Initially, the proportions of uncontrolled BP in the nicardipine group were higher than those in the control; however, it then became lower at all time intervals 8 hours after delivery. The proportions of patients who received additional antihypertensive agents and the median cumulative dosages were lower in the nicardipine group than in the control. Continuous infusion of nicardipine can help manage uncontrolled BP during the postpartum period.

## 1. Introduction

Hypertensive disorders of pregnancy remain one of the leading causes of maternal and perinatal mortality, with an incidence of nearly 10%.^[1–3]^ Guidelines recommend controlling blood pressure (BP) with antihypertensive medication to prevent devastating complications such as intracranial hemorrhage, renal injury, and cardiopulmonary compromise when the persistent elevation of systolic BP ≥ 160 mm Hg or diastolic BP ≥ 110 mm Hg is present in pregnant women diagnosed with hypertensive disorders.^[4–7]^ However, the target BP range to maintain sufficient perfusion to the uterus during pregnancy should not be lowered below a systolic BP of 140 to 150 mm Hg and a diastolic BP of 90 to 100 mm Hg.^[8,9]^ While termination of pregnancy is the ultimate treatment for preeclampsia (PE) and other hypertensive disorders of pregnancy, BP does not normalize immediately after giving birth and may sometimes worsen for a certain period of time.^[10–12]^ Therefore, close monitoring and control of BP is mandatory for reducing maternal complications even after delivery.

Nicardipine, a commonly used antihypertensive medication, is a potent vasodilator, which acts as a calcium channel blocker (CCB) on the vascular smooth muscle wall.^[13–15]^ Nicardipine selectively responds to coronary and cerebral vessels over the systemic circulation, resulting in increased cerebral blood flow and oxygen delivery, with less inotropic effects on the myocardium.^[16,17]^ Thus, nicardipine has been used widely for managing acute severe hypertension in patients with cerebrovascular accidents and postoperative BP control after cardiac or neurovascular surgery.^[16,18]^ Previous studies on the efficacy of nicardipine in women with PE showed promising data that nicardipine may be equivalent or better than other antihypertensive agents in pregnancy, such as hydralazine and beta blockers, while ensuring maternal and fetal safety.^[16,18–23]^ Moreover, during the postpartum period, the target BP can be obtained by taking advantage of nicardipine without fear of causing side effects to the fetus. No studies have been conducted to determine whether the continuous infusion of nicardipine during the postpartum period is useful for the control of severe hypertension, compared to other conventional antihypertensive agents. This study aimed to assess the effectiveness of the continuous nicardipine infusion for the management of uncontrolled BP during the postpartum period.

## 2. Methods

### 2.1. Study design and population

A retrospective cohort study was performed on consecutive pregnant women who had been diagnosed with hypertensive disorders of pregnancy (chronic hypertension, gestational hypertension, PE, superimposed PE, and eclampsia) and had delivered after 24 weeks of gestation due to indications of maternal hypertensive diseases at Seoul National University Bundang Hospital from January 2018 to December 2020.

We used the definitions for each hypertensive disorder of pregnancy according to the guidelines of the American College of Obstetricians and Gynecologists.^[2,24]^ When hypertension was diagnosed before pregnancy, the patient was classified as having chronic hypertension. Gestational hypertension was diagnosed when BP elevation was found after 20 weeks of gestation without proteinuria or other systemic findings. PE was defined as hypertension in association with one of the following: proteinuria or thrombocytopenia (cell counts < 100,000/mL), impaired liver function (2 folds elevation of liver enzymes), progressive renal insufficiency (serum creatinine > 1.1 mg/dL or twice the previous value), pulmonary edema, or new-onset cerebral symptoms or visual disturbance. Superimposed PE was defined as the development of PE in patients already diagnosed with chronic hypertension. If seizures were present in a woman with known PE or other hypertensive disorders, eclampsia was diagnosed.

**Figure 1.**
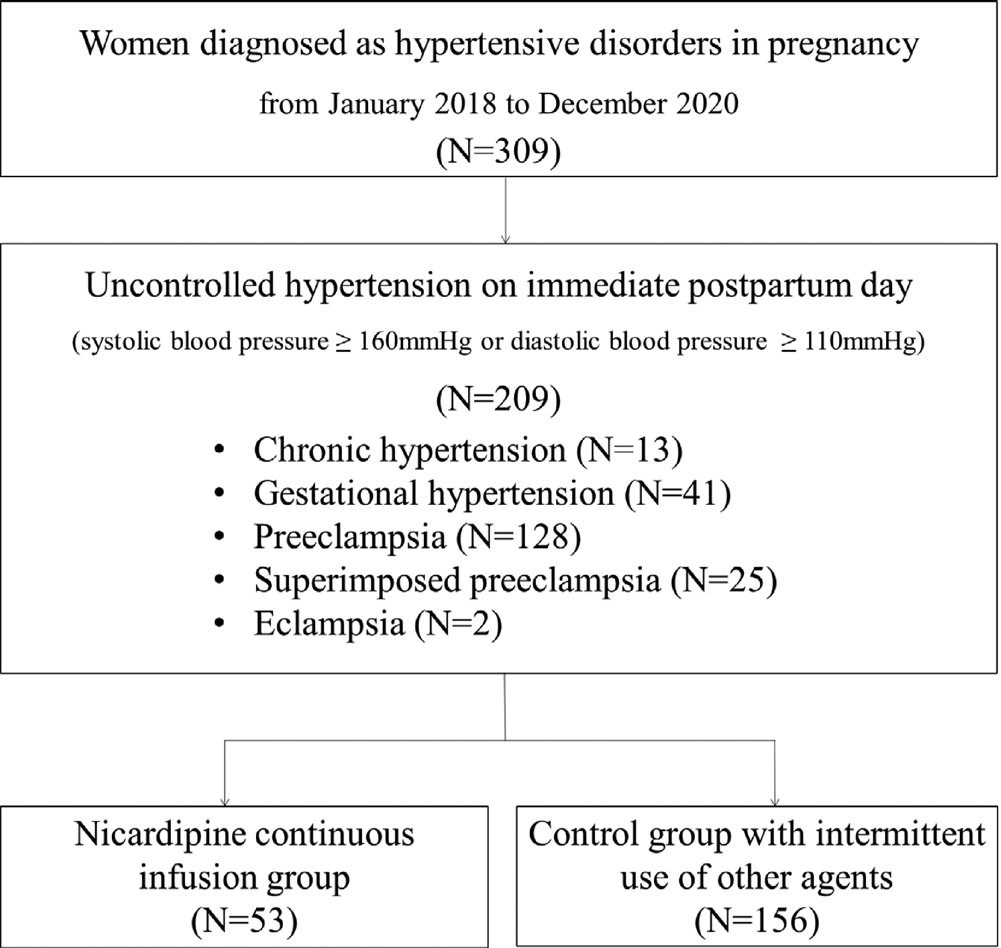
Flow chart of the study population.

Among the 309 patients, 209 (approximately 68%) presented with uncontrolled BP with systolic BP ≥ 160 mm Hg or diastolic BP ≥ 110 mm Hg on the day of delivery (Fig. 1). The study population was divided into 2 groups: patients who received a continuous infusion of nicardipine at the time of initial detection for high BP and those controlled by other antihypertensive medications intermittently administered for every moment of high BP measured. The patient assignment to continuous nicardipine group was implemented; When BP exceeded a severe range (160/110 mm Hg) at least 2 consecutive times during delivery, regardless of whether the patient had undergone vaginal delivery or cesarean section, or; When a patient diagnosed with PE showed at least 1 severe feature. In addition, all patients received magnesium sulfate infusion for seizure prophylaxis.

Maternal characteristics, all the measured BP data, other vital signs, the types and numbers of administered medications, and the cumulative doses were evaluated through electronic medical chart review. All BP data for 28 hours (from 4 hours before delivery to 24 hours after delivery) were analyzed at 4-hour intervals. The highest BP data and the proportion of uncontrolled BP defined as a systolic BP of ≥ 160 mm Hg or a diastolic BP of ≥ 110 mm Hg in each time interval were extracted from each patient. The symptoms considered as side effects (e.g., nausea, dizziness, palpitation, headache, or flushing sense) from medications including antihypertensive agents and others, including magnesium sulfate, were also checked. The study protocol was approved by the Institutional Review Board of the Seoul National University Bundang Hospital (No. B-1905-538-102).

At our institution, intravenous (IV) continuous nicardipine infusion was given with the fixed rate of 0.5 mcg/kg/minutes by infusion pump and was discontinued after approximately a day. Other agents used for postpartum BP control were IV bolus of labetalol (a well-known beta blocker) or hydralazine, and they were given instantaneously whenever BP was measured to exceed a systolic BP of > 160 mm Hg or a diastolic BP of > 110 mm Hg. When either labetalol or hydralazine was administered, BP was measured after 15 minutes of resting, and if BP did not subside to the target range, an additional dose or alternative medication was administered. Once the aforementioned BP thresholds were achieved and found to be consistently controlled, the measurement was repeated with longer intervals, such as every 30 minutes or after certain hours.

Patients with a BP between 140/90 mm Hg and 160/110 mm Hg during pregnancy were treated with oral anti-hypertensive medications to reach a target BP less than 140/90 mm Hg. Patients were monitored in the outpatient department unless severe hypertension developed. In the majority of cases, the prescribed first line anti-hypertensive agent was extended-release nifedipine. Patients were instructed to monitor their BP at home. During clinic visits, the initial prescribed dose (30 mg) of nifedipine was escalated up to 120 mg per day. A minority of patients were prescribed amlodipine (5–10 mg) first if this was preferred by the physician. Second line medication involved the addition of oral hydralazine (12.5 mg) to either nifedipine or amlodipine to achieve the target BP.

### 2.2. Statistical analysis

The Mann–Whitney U-test was used to compare continuous variables, while the *χ*^2^ test was used for categorical variables. Statistical significance was set at *P* < .05. The data were analyzed using SPSS 25.0 (IBM, Armonk, NY). A modularized Python code that automatically performs the standardization process (version 3.6, Python Software Foundation) was used to analyze all the data extracted during hospitalization of the whole study population and to understand the trends of BP changes according to the timelines.

## 3. Results

### 3.1. Patient characteristics

The study population consisted of 135 patients diagnosed with PE, 44 with gestational hypertension, 10 with chronic hypertension, 18 with superimposed PE, and 2 with eclampsia (Fig. 1). Among them, approximately one-fourth of the patients (53/209) received continuous infusion of nicardipine immediately postpartum, while others did not.

The demographic and clinical characteristics of the study population are presented in Table 1. The mean gestational age at delivery was significantly earlier in the nicardipine group than in the control group (34.5 vs 35.1 weeks, *P* = .046). Other maternal characteristics did not differ between the 2 groups. The proportions of each type of hypertensive disorder of pregnancy were comparable; however, the rate of eclampsia was higher in the nicardipine group than in the control group (4% vs 0%, *P* = .015). The additional antihypertensive medications used were IV side shots of labetalol and hydralazine. The proportions of patients receiving labetalol and hydralazine for the control group were 14.1% (22/156) and 13.5% (21/156), respectively, and these numbers were higher than those administered additionally to nicardipine in the continuous nicardipine infusion group (9.4% [5/53] and 5.7% [3/53], respectively). The median total dosages of labetalol and hydralazine used in the control group were 20 mg (range 10–70 mg) and 10 mg (range 5–40 mg), respectively. In the nicardipine group, the dosages of labetalol and hydralazine additionally used with nicardipine were 10 mg (range 10–50 mg) and 8 mg (range 5–10 mg), respectively, which were much lower than those in the control group.

**Table 1:** Demographic and clinical characteristics of the study population according to postpartum continuous nicardipine use.

Characteristics	Nicardipine group (N = 53)	Control group (N = 156)	*P* value
Maternal age (yr)	34.1 ± 4.5	34.8 ± 4.1	.536
Nulliparity	73.6% (39/53)	71.2% (111/156)	.734
Multiple pregnancy	9.4% (5/53)	4.5% (7/156)	.181
Body mass index (kg/m^2^)	30.4 ± 5.9	29.6 ± 5.0	.436
Gestational age at delivery (wk)	34.5 ± 2.9	35.1 ± 3.6	**.046**
Cesarean section	75.5% (40/53)	78.8% (123/156)	.608
Use of oral anti-hypertensive medication during pregnancy	37.7% (20/53)	30.1% (47/156)	.305
Extended-release nifedipine	95% (19/20)	87.2% (41/47)	
Amlodipine	10% (2/20)	21.3% (10/47)	
Hydralazine	15% (3/20)	8.5% (4/47)	
Pregestational diabetes mellitus	1.9% (1/53)	4.5% (7/156)	.394
Gestational diabetes mellitus	20.8% (11/53)	10.9% (17/156)	.069
Previous hypertensive disorder of pregnancy†	50% (7/14)	37.8% (17/45)	.416
Current hypertensive disorder			
Gestational hypertension	11.3% (6/53)	22.4% (35/156)	.105
Preeclampsia	69.8% (37/53)	58.3% (91/156)	.078
Superimposed preeclampsia	9.4% (5/53)	12.8% (20/156)	.512
Chronic hypertension	5.7% (3/53)	6.4% (10/156)	.845
Eclampsia	3.8% (2/53)	0% (0/156)	**.015**

Values are presented as mean ± standard deviation or % (n).

† Analyzed only in multiparous women, bold values represent the statistically significant results.

**Figure 2.**
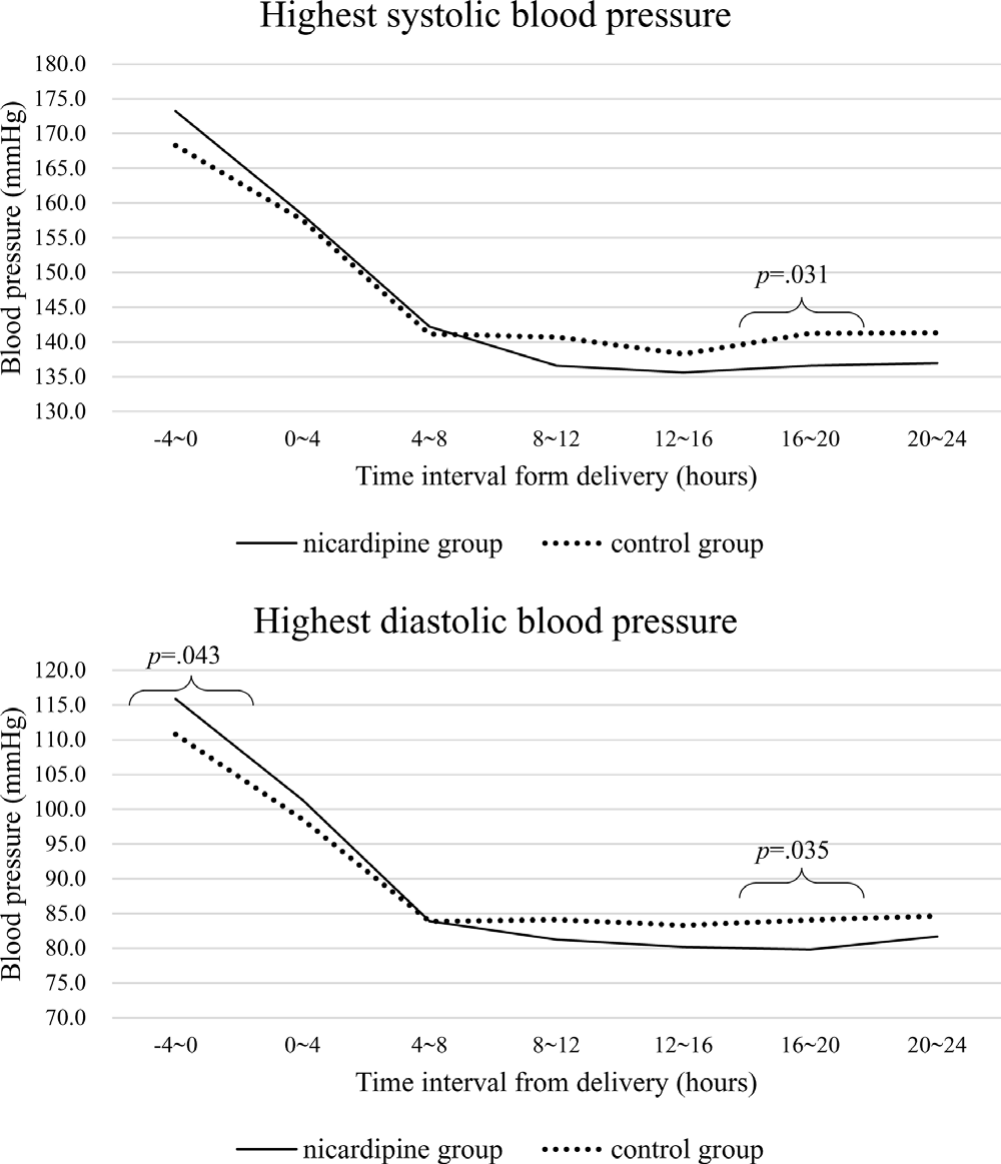
Trends in highest blood pressure described as mean values in each group for 24 hours postpartum.

Table 2 shows a comparison of the averages of highest BP checked in each time interval from 4 hours before delivery to 24 hours postpartum. The mean values of both systolic BP and diastolic BP immediately before delivery were higher in the nicardipine group than in the control group; however, the difference was statistically significant only for the analysis of diastolic BP (116 vs 111 mm Hg, *P* = .043). Up to 8 hours after delivery, there was no significant difference between the 2 groups. After 8 to 12 hours following delivery, the mean values of both systolic and diastolic BP were lower in the nicardipine group than in the control group. Subsequently, in the time interval of 16 to 20 hours following delivery, the averages of both systolic BP and diastolic BP were significantly lower in the nicardipine group than in the control group (137/80 vs 141/84 mm Hg, *P* = .031 for systolic BP, *P* = .035 for diastolic BP). The trends in BP control for the 2 groups are presented in Figure 2.

**Table 2:** Comparison of the highest blood pressure during postpartum 24 hours according to the use of continuous nicardipine infusion.

Time interval from delivery (h)	Nicardipine group (N = 53)		Control group (N = 156)		*P* value	
	Systolic BP	Diastolic BP	Systolic BP	Diastolic BP	*P* value^a^	*P* value^b^
–4 to 0	173.2 ± 16.9	115.9 ± 17.0	168.3 ± 16.5	110.8 ± 17.1	.076	**.043**
0 to 4	158.3 ± 10.7	101.3 ± 10.3	157.6 ± 15.1	98.6 ± 13.6	.995	.068
4 to 8	142.2 ± 12.5	83.9 ± 10.4	141.1 ± 14.5	83.9 ± 12.3	.622	.996
8 to 12	136.6 ± 13.9	81.3 ± 11.0	140.7 ± 13.6	84.1 ± 11.4	.058	.174
12 to 16	135.6 ± 15.5	80.2 ± 13.2	138.3 ± 14.6	83.3 ± 12.5	.228	.133
16 to 20	136.6 ± 13.7	79.8 ± 11.3	141.2 ± 13.8	84.1 ± 11.2	**.031**	**.035**
20 to 24	137.0 ± 15.5	81.7 ± 14.7	141.3 ± 15.4	84.6 ± 10.8	.056	.139

Values are presented as mean ± standard deviation.

BP = blood pressure.

a *P*-value of comparison in systolic blood pressure between 2 groups.

b *P*-value of comparison in diastolic blood pressure between 2 groups, bold values represent the statistically significant results.

**Figure 3.**
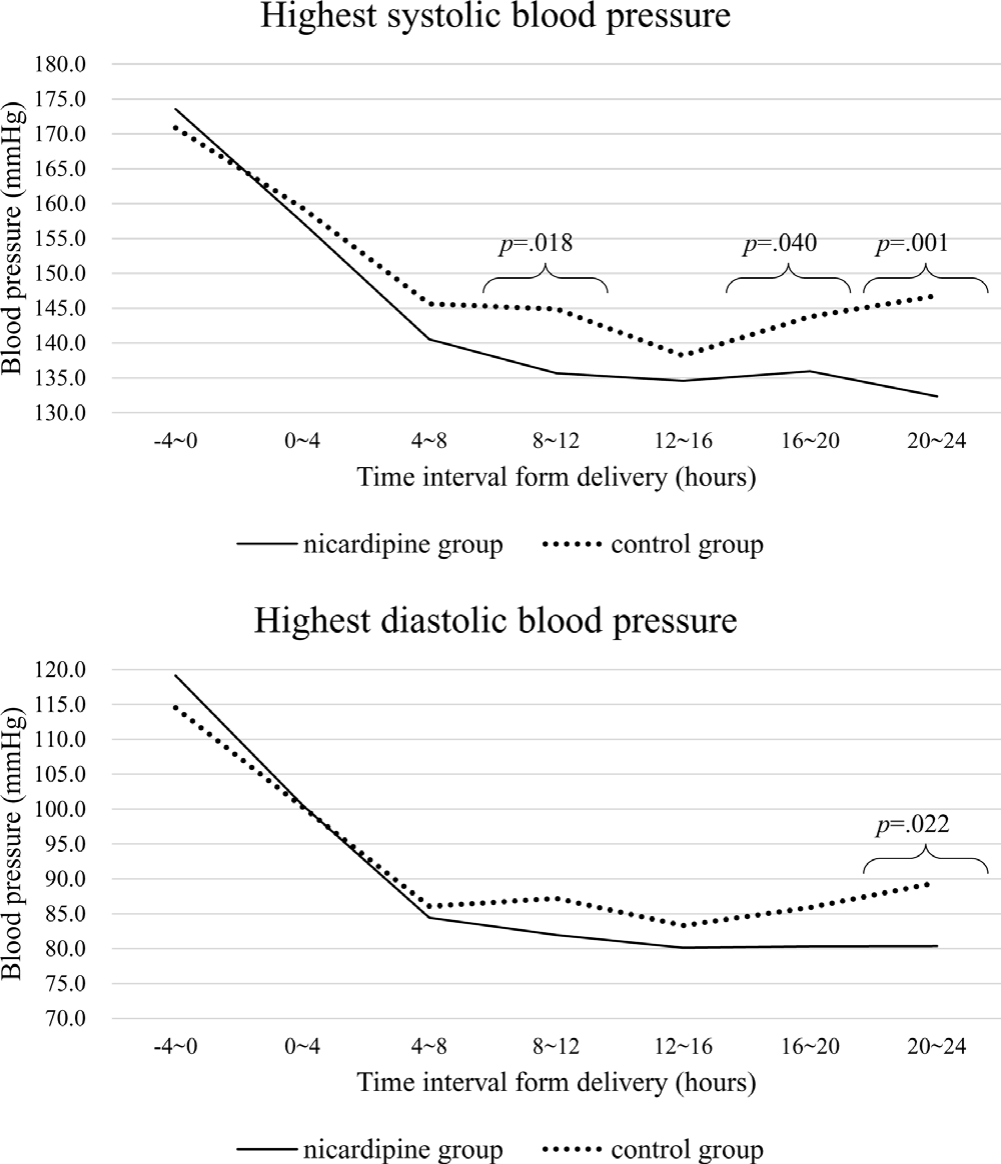
Trends in highest blood pressure described as mean values in each group for 24 hours postpartum among patients with oral anti-hypertensive medication.

Table 3 demonstrates subgroup analysis of the average highest BP in patients taking oral anti-hypertensive medication. A similar BP lowering pattern is shown in relation to the use of continuous nicardipine infusion and time intervals after delivery. The mean values of both systolic and diastolic BP were lower in the nicardipine group than in the control throughout all time intervals, with the exception of the first interval before delivery. Between 20 and 24 hours following delivery, both systolic and diastolic BP were significantly lower in the nicardipine group than in the control group (132/80 vs 146/89 mm Hg). The trends of BP control in the subgroup with oral anti-hypertensive medication are depicted in Figure 3.

**Table 3:** Comparison of the highest blood pressure during the first 24 hours postpartum in patients with oral anti-hypertensive medication during pregnancy, according to the use of continuous nicardipine infusion.

Time interval from delivery (h)	Nicardipine group (N = 20)		Control group (N = 47)		*P* value	
	Systolic BP (mm Hg)	Diastolic BP (mm Hg)	Systolic BP (mm Hg)	Diastolic BP (mm Hg)	*P* value^a^	*P* value^b^
–4 to 0	173.6 ± 11.1	119.2 ± 19.8	170.9 ± 18.6	114.5 ± 20.6	.305	.406
0 to 4	157.3 ± 9.3	100.6 ± 9.4	159.4 ± 15.9	100.3 ± 13.8	.569	.656
4 to 8	140.5 ± 8.9	84.4 ± 6.4	145.6 ± 17.3	86.0 ± 14.9	.374	.532
8 to 12	135.7 ± 12.3	81.9 ± 10.7	144.9 ± 15.6	87.2 ± 12.4	**.018**	.097
12 to 16	134.6 ± 12.0	80.1 ± 14.4	138.2 ± 13.6	83.3 ± 13.1	.277	.504
16 to 20	135.9 ± 13.6	80.3 ± 11.5	143.8 ± 13.9	85.9 ± 11.8	**.040**	.116
20 to 24	132.4 ± 13.4	80.4 ± 13.1	146.8 ± 13.5	89.5 ± 9.4	**.001**	**.022**

Values are presented as mean ± standard deviation.

BP = blood pressure.

a *P*-value of comparison in systolic blood pressure between 2 groups.

b *P*-value of comparison in diastolic blood pressure between 2 groups.

**Figure 4.**
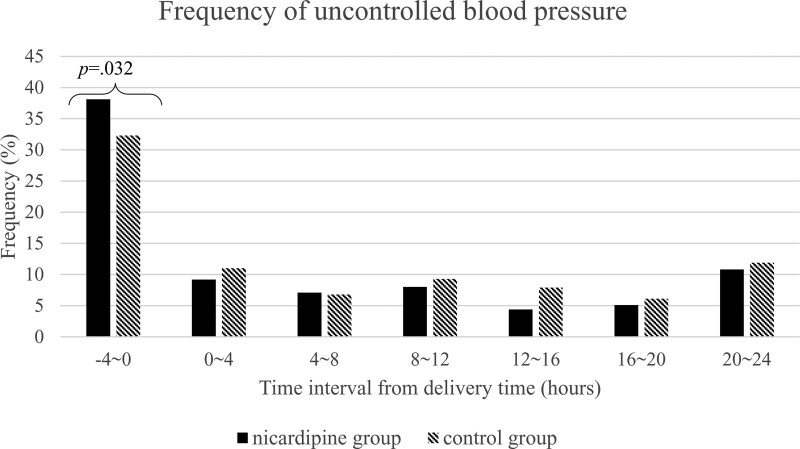
Frequency of uncontrolled blood pressure (BP) (systolic BP ≥ 160 mm Hg or diastolic BP ≥ 110 mm Hg) for 24 hours postpartum, stratified by 4-hour interval. BP = blood pressure.

The numbers of uncontrolled BP (defined as a systolic BP of > 160 mm Hg or a diastolic BP of > 110 mm Hg) detected for each group are shown in Figure 4. Before delivery, the nicardipine group showed higher proportions of uncontrolled BP detection than the control group (38% vs 32%, *P* = .032). The rates of uncontrolled BP detection during the postpartum period were lower in the nicardipine group than in the control group at all the time intervals from 8 hours following delivery, even though the difference did not reach statistical significance.

## 4. Discussion

According to the detection of BP for each 4-h-interval, the nicardipine group had both lower systolic BP and diastolic BP than the control group in the postpartum period, especially after 8 hours following delivery. The frequency of severely high BP detected in the postpartum period was lower in the nicardipine group than in the control group, and the trend graph demonstrated the possibility of a more stable antihypertensive effect of continuous nicardipine infusion than the control group, which followed the protocol using intermittent administration for each detection of uncontrolled high BP.

These results could be meaningful since the nicardipine group is assumed to have more severe patients or severe clinical features compared with the control group. In fact, the proportions of currently diagnosed hypertensive disorders and other maternal characteristics were not different; however, the gestational age at delivery was significantly earlier in the nicardipine group than the control group (34.5 vs 35.1 weeks, *P* = .046), implying that the clinical features of the nicardipine group could have been slightly more emergent than those of the control group. Moreover, the proportion of eclampsia was 4% in the nicardipine group and none in the control group. Although the features were more severe in patients who had received continuous nicardipine infusion, Figures 2 and 3 show that hypertension in the nicardipine group was controlled better than that of the control group with several short-acting shots of other agents.

If the effect to control hypertension is better or at least comparable to other antihypertensive agents, continuous infusion of nicardipine for the acute phase of severely high BP in the postpartum period helps in the management of hypertensive disorders in pregnancy. In clinical settings, close monitoring with repeated administration of short active agents and frequent measurements of vital signs thereafter for postpartum patients is a remarkable burden for medical facilities in terms of costs and labor of human resources. In this study, continuous infusion of CCB during the postpartum period in patients who had given birth and still presented with uncontrolled hypertension could be an alternative for intermittent use of antihypertensive agents at the sight of detection for high BP and could be used as a routine protocol for some indicated high-risk pregnancies, expecting to have a long time to recover from hypertension developed during pregnancy, even after delivery.

Severe hypertension that develops during the postpartum period is exacerbated by various iatrogenic conditions (e.g., volume retention, sympathomimetic activation, and vasoconstriction by ergonovine).^[8,25]^ Acute and sudden onset of uncontrolled BP can lead to endothelial damage to multiple organs, cerebral edema sometimes accompanied by brain injury, and potentially maternal death.^[8,20,25–27]^ Thus, severe hypertension is an emergent condition for all pregnant women diagnosed with PE or other hypertensive disorders. Nevertheless, the postpartum state is likely to be neglected or is monitored less intensively than during pregnancy because termination of pregnancy is a well-known treatment of choice. A previous study pointed out that one of the factors associated with delayed treatment for hypertensive emergencies was overnight severe hypertension occurring between 10 PM and 6 AM.^[28]^ During the night, striking BP or symptoms of hypertension could be missed. Continuous infusion of nicardipine can prevent these risks and, thus, could be a complementary method to keep BP stable after delivery.

The antihypertensive mechanism of CCBs is far more suitable than other agents from the perspective of the pathophysiology of PE and other hypertensive disorders specified in pregnancy. In patients with PE, cardiac output is decreased, and intravascular volume expansion is restricted due to elevated systemic vascular resistance. This systematic change would impact decreased renal blood flow, lowered glomerular infiltration rate, and eventually oliguria.^[9]^ Nicardipine has been proven to have advantages in increasing coronary blood flow and cardiac pumping activity. In addition, nicardipine improves glomerular infiltration rate by lowering renovascular resistance.^[29]^ Therefore, nicardipine can act as an effective antihypertensive agent that can provide additional positive effects and manage severe hypertension, especially in women with PE with severe features.

In the subgroup analysis, continuous IV infusion of nicardipine showed a greater anti-hypertensive effect among patients with oral medications. One possible explanation the is pharmacokinetic interaction of CCB. In the study population, the major type of oral anti-hypertensive medication was CCB, in particular, extended-release nifedipine followed by amlodipine. Thus, it is plausible that enhanced pharmacological effects of the combination of dual CCB therapy would result in greater anti-hypertensive effect.^[30]^ In fact, patients receiving oral medication were assumed to have more chronic hypertension compared to patients without oral medication. The rates of superimposed preeclampsia and chronic hypertension were higher in patients with oral medication compared with those without (*P* < .001, 31.3% vs 2.8% for superimposed preeclampsia; *P* < .001, 14.9% vs 2.1% for chronic hypertension). Thus, it could be presumed that the additive effect of nicardipine with either nifedipine or amlodipine was reflected in the significantly greater anti-hypertensive activity, particularly in patients with chronic hypertension.

A few current guidelines recommend continuous IV infusion of nicardipine as a second-line alternative to refractory hypertension, which is not controlled by other initiating agents such as IV bolus of labetalol or hydralazine.^[11,25,26]^ Nicardipine infusion is sometimes used for the antenatal period as well; however, CCBs are not commonly infused during pregnancy because they affect the muscular functions of the uterus and can lower the tone of the extremities or respiratory muscles. Oral CCB agents are used as tocolytics as well as antihypertensive agents during pregnancy.

Although few adverse maternal reactions have been reported for the use of nicardipine during pregnancy compared with that for the use of hydralazine, labetalol, or ketanserin,^[19]^ there are still concerns regarding its use during pregnancy and even after delivery. In this study, 11 patients in the nicardipine group complained of minor side effects, including nausea, palpitation, headache, and flushing sensation. There were no complaints or conditions complicated with the administration of nicardipine; however, the possible side effects caused by nicardipine, especially when magnesium sulfate was administered together, have not been clearly studied in pregnant women. There are some conflicting data from animal studies showing that nicardipine could be associated with uteroplacental hypoperfusion due to abrupt hypotension or acidosis of the fetus.^[31–33]^ Few studies have reported the synergetic effects of the concurrent use of nicardipine and magnesium sulfate for conditions such as hypotension and myocardial depression from disturbance of calcium influx.^[4,17,34]^ In this study, the condition of all patients was severe enough to use postpartum magnesium sulfate for eclampsia prevention; however, none of the patients had the aforementioned adverse effects. However, concomitant use of nicardipine and magnesium sulfate should be monitored carefully when a maternal cardiovascular problem is compromised.

To the best of our knowledge, this is the first study to evaluate the effectiveness of continuous IV infusion of nicardipine during the postpartum period accompanied by severe hypertension in patients diagnosed with PE and hypertensive diseases. This study has some limitations, such as the small sample size and the retrospective nature; however, the implications of these selected patients restricted to severe PE or hypertensive disorders of pregnancy with severe hypertension at a single center with unified and consistent protocols in clinical practice can be the initiation for the use of nicardipine infusion for certain indications. Future studies should evaluate the effects or usefulness of continuous infusion of nicardipine for persistent postpartum hypertension and evaluate its safety.

## Author contribution

Authors declare that they participated in the following roles and that they have read and approved the final version.

**Conceptualization:** Min Kyung Kim, Hyeon Ji Kim, Jee Yoon Park.

**Data curation:** Min Kyung Kim, Ki Roong Choe, Da Eun Jeong.

**Formal analysis:** Min Kyung Kim, Ki Roong Choe, Kyong-No Lee, Iseop Cho, Hyeon Ji Kim.

**Writing – original draft:** Min Kyung Kim, Ki Roong Choe, Da Eun Jeong, Kyong-No Lee, Iseop Cho, Hyeon Ji Kim.

**Writing – review & editing:** Hyeon Ji Kim, Jee Yoon Park.

## References

[1] Khedagi AM Bello NA. Hypertensive disorders of pregnancy. Cardiol Clin. 2021;39:77–90. 33222817 10.1016/j.ccl.2020.09.005 PMC7720658

[2] Agrawal A Wenger NK. Hypertension during pregnancy. Curr Hypertens Rep. 2020;22:64. 32852628 10.1007/s11906-020-01070-0

[3] Ukah UV De Silva DA Payne B. Prediction of adverse maternal outcomes from pre-eclampsia and other hypertensive disorders of pregnancy: a systematic review. Preg Hypertens. 2018;11:115–23. 10.1016/j.preghy.2017.11.006 29198742

[4] Sinkey RG Battarbee AN Bello NA. Prevention, diagnosis, and management of hypertensive disorders of pregnancy: a comparison of international guidelines. Curr Hypertens Rep. 2020;22:66. 32852691 10.1007/s11906-020-01082-w PMC7773049

[5] Turner K Hameed AB. Hypertensive disorders in pregnancy current practice review. Curr Hypertens Rev. 2017;13:80–8. 28554307 10.2174/1573402113666170529110024

[6] ACOG Practice Bulletin No. 202. Gestational hypertension and preeclampsia. Obstet Gynecol. 2019;133:1. 10.1097/AOG.0000000000003018 30575675

[7] American College of Obstetricians and Gynecologists’ Committee on Practice Bulletins–Obstetrics. ACOG practice bulletin No. 203: chronic hypertension in pregnancy. Obstet Gynecol. 2019;133:e26–50. 30575676 10.1097/AOG.0000000000003020

[8] Too GT Hill JB. Hypertensive crisis during pregnancy and postpartum period. Semin Perinatol. 2013;37:280–7. 23916027 10.1053/j.semperi.2013.04.007

[9] Alexander JM Wilson KL. Hypertensive emergencies of pregnancy. Obstet Gynecol Clin North Am. 2013;40:89–101. 23466139 10.1016/j.ogc.2012.11.008

[10] Hauspurg A Countouris ME Catov JM. Hypertensive disorders of pregnancy and future maternal health: how can the evidence guide postpartum management? Curr Hypertens Rep. 2019;21:96. 31776692 10.1007/s11906-019-0999-7 PMC7288250

[11] Sharma KJ Kilpatrick SJ. Postpartum hypertension: etiology, diagnosis, and management. Obstet Gynecol Surv. 2017;72:248–52. 28426127 10.1097/OGX.0000000000000424

[12] ACOG committee opinion no.767. Emergent therapy for acute-onset, severe hypertension during pregnancy and the postpartum period. ACOG committee opinion. Obstet Gynecol. 2019;133:e174–80. 30575639 10.1097/AOG.0000000000003075

[13] Qi H Qin J Ren L. Efficacy of low-dose nicardipine for emergent treatment of severe postpartum hypertension in maternal intensive care units: an observational study. Preg Hypertens. 2020;21:43–9. 10.1016/j.preghy.2020.04.012 32388119

[14] Hecht JP Richards PG. Continuous-infusion labetalol vs nicardipine for hypertension management in stroke patients. J Stroke Cerebrovasc Dis. 2018;27:460–5. 29092768 10.1016/j.jstrokecerebrovasdis.2017.09.023

[15] Stone ML Kelly J Mistry M. Use of nicardipine after cardiac operations is safe in children regardless of age. Ann Thorac Surg. 2018;105:181–5. 28987396 10.1016/j.athoracsur.2017.05.035

[16] Curran MP Robinson DM Keating GM. Intravenous nicardipine: its use in the short-term treatment of hypertension and various other indications. Drugs. 2006;66:1755–82. 16978041 10.2165/00003495-200666130-00010

[17] Kaplan JA. Clinical considerations for the use of intravenous nicardipine in the treatment of postoperative hypertension. Am Heart J. 1990;119(2 Pt 2):443–6. 2405614 10.1016/s0002-8703(05)80066-3

[18] Peacock WF Hilleman DE Levy PD. A systematic review of nicardipine vs labetalol for the management of hypertensive crises. Am J Emerg Med. 2012;30:981–93. 21908132 10.1016/j.ajem.2011.06.040

[19] Nij Bijvank SW Duvekot JJ. Nicardipine for the treatment of severe hypertension in pregnancy: a review of the literature. Obstet Gynecol Surv. 2010;65:341–7. 20591204 10.1097/OGX.0b013e3181e2c795

[20] Matsuura A Yamamoto T Arakawa T. Management of severe hypertension by nicardipine intravenous infusion in pregnancy induced hypertension after cesarean section. Hypertens Res Pregnancy. 2015;3:28–31.

[21] Elatrous S Nouira S Ouanes Besbes L. Short-term treatment of severe hypertension of pregnancy: prospective comparison of nicardipine and labetalol. Intensive Care Med. 2002;28:1281–6. 12209278 10.1007/s00134-002-1406-3

[22] Hanff LM Vulto AG Bartels PA. Intravenous use of the calcium-channel blocker nicardipine as second-line treatment in severe, early-onset pre-eclamptic patients. J Hypertens. 2005;23:2319–26. 16269975 10.1097/01.hjh.0000188729.73807.16

[23] Nooij LS Visser S Meuleman T. The optimal treatment of severe hypertension in pregnancy: update of the role of nicardipine. Curr Pharm Biotechnol. 2014;15:64–9. 24720593 10.2174/1389201015666140330194722

[24] Hypertension in pregnancy. Report of the American college of obstetricians and gynecologists’ task force on hypertension in pregnancy. Obstet Gynecol. 2013;122:1122–31. 24150027 10.1097/01.AOG.0000437382.03963.88

[25] Gestational hypertension and preeclampsia. ACOG Practice Bulletin, Number 222. Obstet Gynecol 2020;135:e237–60. 32443079 10.1097/AOG.0000000000003891

[26] Committee Opinion No. 623: emergent therapy for acute-onset, severe hypertension during pregnancy and the postpartum period. Obstet Gynecol. 2015;125:521–5. 25611642 10.1097/01.AOG.0000460762.59152.d7

[27] Hammer ES Cipolla MJ. Cerebrovascular dysfunction in preeclamptic pregnancies. Curr Hypertens Rep. 2015;17:64. 26126779 10.1007/s11906-015-0575-8 PMC4752117

[28] Kantorowska A Heiselman CJ Halpern TA. Identification of factors associated with delayed treatment of obstetric hypertensive emergencies. Am J Obstet Gynecol. 2020;223:250.e1–e11. 10.1016/j.ajog.2020.02.009 32067968

[29] Sorkin EM Clissold SP. Nicardipine. A review of its pharmacodynamic and pharmacokinetic properties, and therapeutic efficacy, in the treatment of angina pectoris, hypertension and related cardiovascular disorders. Drugs. 1987;33:296–345. 3297616 10.2165/00003495-198733040-00002

[30] Alviar CL Devarapally S Nadkarni GN. Efficacy and safety of dual calcium channel blockade for the treatment of hypertension: a meta-analysis. Am J Hypertens. 2013;26:287–97. 23382415 10.1093/ajh/hps009

[31] Holbrook RH Jr Voss EM Gibson RN. Ovine fetal cardiorespiratory response to nicardipine. Am J Obstet Gynecol. 1989;161:718–21. 2782355 10.1016/0002-9378(89)90388-8

[32] Parisi VM Salinas J Stockmar EJ. Fetal vascular responses to maternal nicardipine administration in the hypertensive EWE. Am J Obstet Gynecol. 1989;161:1035–9. 2801819 10.1016/0002-9378(89)90779-5

[33] Janower S Carbonne B Lejeune V. [Acute pulmonary edema during preterm labor: role of nicardipine tocolysis (three cases)]. [OEdème pulmonaire aigu lors d’une menace d’accouchement prématuré: rôle de la tocolyse par la nicardipine: à propos de 3 observations]. J Gynecol Obstet Biol Reprod (Paris). 2005;34:807–12. 16319773 10.1016/s0368-2315(05)82958-8

[34] Carles G Helou J Alassas N. [Complications of association magnesium sulfate with nicardipine during preeclampsia: report of 2 cases]. [Complications de l’association sulfate de magnésium et nicardipine au cours de la prééclampsie: à propos de 2 cas]. Gynecol Obstet Fertil. 2012;40:614–6. 22981126 10.1016/j.gyobfe.2012.07.003

